# Train duration and inter-train interval determine the direction and intensity of high-frequency rTMS after-effects

**DOI:** 10.3389/fnins.2023.1157080

**Published:** 2023-07-05

**Authors:** Jingna Jin, Xin Wang, He Wang, Ying Li, Zhipeng Liu, Tao Yin

**Affiliations:** ^1^Institute of Biomedical Engineering, Chinese Academy of Medical Sciences and Peking Union Medical College, Tianjin, China; ^2^Neuroscience Center, Chinese Academy of Medical Sciences, Beijing, China

**Keywords:** high-frequency repetitive transcranial magnetic stimulation, metaplasticity, train duration, inter-train interval, motor evoked potential, electroencephalography

## Abstract

**Background and objective:**

It has been proved that repetitive transcranial magnetic stimulation (rTMS) triggers the modulation of homeostatic metaplasticity, which causes the effect of rTMS to disappear or even reverse, and a certain length of interval between rTMS trains might break the modulation of homeostatic metaplasticity. However, it remains unknown whether the effects of high-frequency rTMS can be modulated by homeostatic metaplasticity by lengthening the train duration and whether homeostatic metaplasticity can be broken by prolonging the inter-train interval.

**Methods:**

In this study, 15 subjects participated in two experiments including different rTMS protocols targeting the motor cortex. In the first experiment, high-frequency rTMS protocols with different train durations (2 s and 5 s) and an inter-train interval of 25 s were adopted. In the second experiment, high-frequency rTMS protocols with a train duration of 5 s and different inter-train intervals (50 s and 100 s) were adopted. A sham protocol was also included. Changes of motor evoked potential amplitude acquired from electromyography, power spectral density, and intra-region and inter-region functional connectivity acquired from electroencephalography in the resting state before and after each rTMS protocol were evaluated.

**Results:**

High-frequency rTMS with 2 s train duration and 25 s inter-train interval increased cortex excitability and the power spectral density of bilateral central regions in the alpha frequency band and enhanced the functional connectivity between central regions and other brain regions. When the train duration was prolonged to 5 s, the after-effects of high-frequency rTMS disappeared. The after-effects of rTMS with 5 s train duration and 100 s inter-train interval were the same as those of rTMS with 2 s train duration and 25 s inter-train interval.

**Conclusion:**

Our results indicated that train duration and inter-train interval could induce the homeostatic metaplasticiy and determine the direction of intensity of rTMS after-effects, and should certainly be taken into account when performing rTMS in both research and clinical practice.

## 1. Introduction

Repetitive transcranial magnetic stimulation (rTMS) is a non-invasive brain stimulation technology that can modulate cortical activity using time-varying magnetic fields, and the effects of rTMS can outlast the stimulation time (Hayashi et al., 2004; Thut and Pascual-Leone, 2010). Furthermore, rTMS can modulate not only the targeted cortex but also distal brain regions that have functional connections with the targeted cortex (Di Lazzaro et al., 2011; Jin et al., 2017). As an effective tool to improve motor function and cognitive learning, rTMS has been used in the treatment of neuropsychiatric disorders (Lewis, 2018; Li et al., 2018; Lefaucheur et al., 2020).

Many parameters of rTMS protocols can be adjusted, including stimulation intensity, stimulation frequency, stimulation duration, train duration, and inter-train interval. By adjusting these stimulation parameters, rTMS can modulate the neural activity in both directions and improve brain function (Arai et al., 2007; Taylor and Loo, 2007; Jung et al., 2008). Previously, it was thought that the excitatory or inhibitory properties of rTMS protocols are dependent on the stimulation frequency; specifically, low-frequency (≤1 Hz) rTMS decreases the excitability of the motor cortex, and high-frequency (≥5 Hz) rTMS induces facilitatory effects (Houdayer et al., 2008; Di Lazzaro et al., 2011). However, many recent studies have confirmed that the dichotomy of the rTMS effect based solely on stimulation frequency is not reasonable (Rothkegel et al., 2010; Wischnewski and Schutter, 2015). By adjusting stimulation parameters, such as inter-train interval and stimulation duration, high-frequency rTMS can reduce the excitability of the motor cortex, low-frequency rTMS can improve the excitability of the motor cortex, and the after-effects of rTMS on the motor cortex can be eliminated. These observations suggest that it is important to research the after-effects of rTMS stimulation parameters on the brain in order to apply rTMS reasonably.

The effects of rTMS modulating the brain is also affected by the instantaneous state of neural oscillation. It was reported that the instantaneous phase of mu-rhythm in motor area might be reflect the instantaneous state of brain neuron, and the amplitude of MEP induced by TMS at different phase of mu-rhythm was significantly different (Hussain et al., 2019). In 2018, Zrenner et al. (2018) developed a brain-state dependent TMS based on mu-rhythm phase, and demonstrated causally the brain-state-dependent effect of rTMS. On the other hand, The after-effect of rTMS stimulation parameters on brain activity is affected by homeostatic metaplasticity, an important mechanism for maintaining overall synaptic weight and firing rate in a neuronal network within the physiological range (Ziemann and Siebner, 2008; Karabanov et al., 2015; Müller-Dahlhaus and Ziemann, 2015). For example, high-frequency rTMS without inter-train interval does not increase cortical excitability (Rothkegel et al., 2010). Additionally, doubling the stimulation duration of intermittent theta burst stimulation (iTBS) and continuous theta burst stimulation (cTBS) respectively decreased and increased cortical excitability (Gamboa et al., 2010, 2011; Murakami et al., 2012). These findings in humans can be explained by the homeostatic metaplasticity mechanism, that is, if rTMS with a long stimulation duration is applied, the first epoch of the rTMS pulses modulate the brain activities to a specific state, and the effects of the second epoch of the rTMS pulses on brain activities are restricted or reversed because of homeostatic metaplasticity.

An animal study using rat hippocampal slices showed that doubling the stimulation duration of iTBS induced homeostatic plasticity, but additive long-term potentiation effects occurred if a delay of 1 h was set between iTBS sessions (Kramár et al., 2012). This phenomenon was also found in subsequent studies with human subjects, indicating that the stimulation interval of rTMS could make the rTMS after-effect break the homeostatic plasticity, and even produce a stronger effect (Opie et al., 2017; Tse et al., 2018). Hence, the stimulation interval and the stimulation duration during high-frequency rTMS might be the crucial factors determining the direction and intensity of neuroplastic changes.

The after-effect of high-frequency rTMS refers to the accumulated effects of each stimulation train. In theory, if the effect of a single stimulation train of the high-frequency rTMS is to increase the cortical excitability, then the after-effect of high-frequency rTMS would be the augmentation of cortical excitability. If the duration of a single stimulation train of the high-frequency rTMS is prolonged, homeostatic metaplasticity might be triggered, resulting in the disappearance or reversal of rTMS after-effects. Furthermore, by prolonging the inter-train intervals of a high-frequency rTMS protocol, the effect of rTMS on cortical excitability can be increased or reproduced. By understanding the homeostatic metaplasticity characteristics of the brain induced by train duration and inter-train interval, brain-state dependent TMS protocols based on homeostatic metaplasticity would be developed in the future, and the effect of rTMS on the brain would be further enhanced. However, the effects of train duration and inter-train interval in high-frequency rTMS have been largely overlooked. Therefore, we hypothesized that (1) prolonging the train duration of high-frequency rTMS might result in the disappearance or reversal of excitatory effects and (2) the excitatory effect can be maintained by extending the inter-train intervals.

In our study, we designed two experiments involving 10 Hz high-frequency rTMS. In the first experiment, the train durations were set to 2 s and 5 s, and the inter-train interval was 25 s. Several studies have proved that high-frequency rTMS with 2 s train duration increases the excitability of the motor cortex (Di Lorenzo et al., 2013; Cha and Hwang, 2022). In the second experiment, considering that too long train duration might lead to safety problems, and it was safe to not exceed 5 s for train duration according to TMS safety guidelines when stimulation frequency is 10 Hz (Rossi et al., 2009, 2020). And there were study showing that rTMS with 5 s train duration could not enhance the cortical excitability (Jung et al., 2008; Huang et al., 2017). So, in our study, the train duration was set to 5 s, and the inter-train interval was prolonged to 50 s and 100 s. The motor evoked potential (MEP) and electroencephalography (EEG) in the resting closed-eye state were measured before and after rTMS. The changes of cortical excitability, power spectral density, and intra-region and inter-region functional connectivity were observed.

## 2. Methods

### 2.1. Subjects

Fifteen healthy subjects participated in the study (mean age 25.07 ± 1.79 years; 8 women). All subjects were screened for any contraindications to TMS (Nyffeler and Müri, 2010). None had been diagnosed with any significant neurological disorder, had any implanted metallic electrical device, or had taken any medication in the 7 days before their participation in the experiment. All subjects were right-handed according to the Edinburgh Handedness Inventory (Oldfield, 1971) and naïve to rTMS, and all of them provided written informed consent in accordance with the Declaration of Helsinki. The Ethics Committee of the Institute of Biomedical Engineering, Chinese Academy of Medical Sciences and Peking Union Medical College, approved the study.

### 2.2. Procedure

A frequency of 10 Hz was chosen for rTMS, as this has been shown to be effective and has been widely used in the research on human brain function and the treatment of disease. Based on the rTMS safety guidelines (Chen et al., 1997; Wassermann, 1998; Rossi et al., 2009, 2020), 10 Hz rTMS at 80% resting motor threshold (RMT) was performed, and 1,200 pulses were delivered for each rTMS protocol. All experiments were performed at the same time of day (from 9 a.m. to 11 a.m.) to avoid variability due to diurnal effects (Sale et al., 2007; Ridding and Ziemann, 2010). All experiments were performed by an experienced experimenter to ensure consistency.

**Figure 1:**
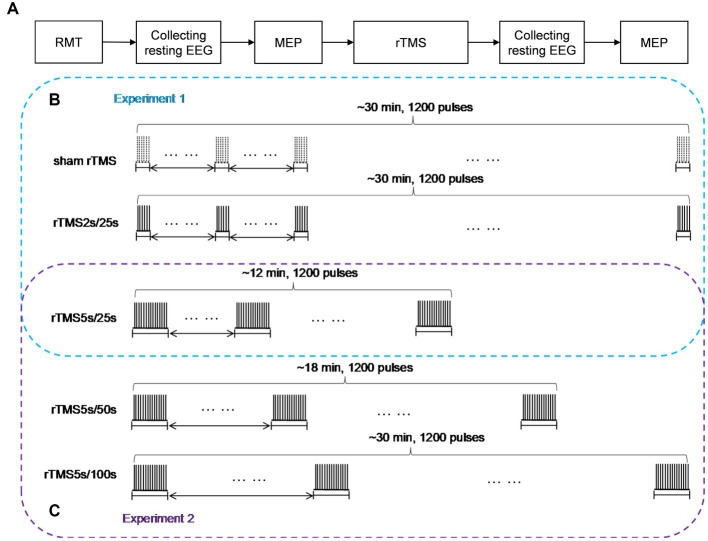
Study design schematic. **(A)** Baseline RMT was assessed before each rTMS protocol, resting EEG in closed eye state, and MEP measurements were obtained before and after 10 Hz rTMS. **(B)** In experiment 1, rTMS protocols with a train duration of either 2 s or 5 s and an inter-train interval of 25 s, a sham protocol were also administered, the order of which was pseudo-randomized and counterbalanced. **(C)** In experiment 2, rTMS protocols with an inter-train interval of either 50 s or 100 s and a train duration of 5 s were administered, the order of which was pseudo-randomized and counterbalanced. The 25 s inter-train interval was adopted in experiment 1. So, we could obtain the effects of rTMS with three different inter-train intervals in experiment 2. rTMS2s/25 s, 2 s train duration and 25 s inter-train interval; rTMS5s/25 s, 5 s train duration and 25 s inter-train interval; rTMS5s/50s, 5 s train duration and 50s inter-train interval; rTMS5s/100 s, 5 s train duration and 100 s inter-train interval.

The experimental paradigm was shown in Figure 1A. At the beginning of each rTMS session, the RMT was measured to prevent RMT fluctuations over time. Before and after each rTMS protocol, EEG was recorded for at least 3 min in the resting state with eyes closed, and MEP was measured through single pulse TMS targeting the motor cortex. After recording the resting state EEG signals after rTMS, we needed switch on the rTMS instrument and set the stimulus parameters required for MEP measurement, so MEP was measured at an interval of 5 min after rTMS. All subjects participated in both experiment 1 and experiment 2. Throughout the experiments, the subjects were not told which rTMS protocol was being used.

Experiment 1

We studied the effects of train duration of high-frequency rTMS on brain activities (Figure 1B). All subjects received three rTMS protocols, administered at least 5 days apart. For each protocol, the inter-train interval was 25 s. One protocol was rTMS with 2 s train duration (total duration: ~30 min), and another protocol was rTMS with 5 s train duration (total duration: ~12 min). A sham rTMS protocol was also adopted, and the pattern of the sham protocol was identical to the rTMS protocol with 2 s train duration. For the sake of convenience, we used intuitive abbreviations for protocols: the abbreviation for rTMS with 2 s train duration and 25 s inter-train interval was rTMS2s/25 s, rTMS with 5 s train duration and 25 s inter-train interval was rTMS5s/25 s. The order in which the protocols were administered was pseudo-randomized and counterbalanced.

Experiment 2

We studied the effects of inter-train interval of high-frequency rTMS on brain activities (Figure 1C). All subjects received two rTMS protocols, administered at least 5 days apart. One protocol had an inter-train interval of 50 s (total duration: ~18 min), and the other had an inter-train interval of 100 s (total duration: ~30 min). Both protocols had a train duration of 5 s. For the sake of convenience, we used intuitive abbreviations for protocols: the abbreviation for rTMS with 5 s train duration and 50 s inter-train interval was rTMS5s/50s, rTMS with 5 s train duration and 100 s inter-train interval was rTMS5s/100 s. The order in which the protocols were administered was pseudo-randomized and counterbalanced. It should be noted that the 25 s inter-train interval was adopted in experiment 1. So, we could obtain the effects of rTMS with three different inter-train intervals.

### 2.3. TMS

TMS was generated through a figure-eight shaped coil (70 mm standard coil, Mastic, Whitland, United Kingdom) connected to a Magnetic Rapid^2^ stimulator (The Magstim Company, Whitland, United Kingdom). For all TMS in our study, the stimulation was guided by a neuronavigation system (Brainsight, Rogue Inc., United Kingdom) to precisely define the neuroanatomical target of TMS from a T1-weighted magnetic resonance image of the subject’s brain. The coil was held tangentially to the skull with the handle pointing backward, at an angle of 45° to the sagittal plane, such that an anterior–posterior current flow, followed by a posterior–anterior current flow (AP-PA), was induced in the underlying cortex. The coil was held over the hand area of the left motor cortex. The scalp position resulting in the most consistent and largest MEP in the first dorsal interosseous muscle (i.e., the motor “hotspot”) was determined and used throughout the session. The RMT was then determined as the minimum intensity necessary to elicit at least 5 out of 10 continuous MEPs with a peak-to-peak amplitude greater than 50 μV while the target muscle was relaxed.

### 2.4. Electromyography

The surface EMG was recorded from the first dorsal interosseous muscle via Ag/AgCl electrodes in a belly-tendon montage (Myoquick Matrix Line-Micromed Srl, Mogliano Veneto, Italy). The ground electrode was placed over the pisiform bone. The sampling rate of the signal was 32,768 Hz. The stimulus intensity was then set to 120% RMT to test the MEP, according to previous literatures (Di Lazzaro et al., 2011; Casula et al., 2014; Pellegrini et al., 2018; Hussain et al., 2019; Thomson et al., 2019). This stimulus intensity was used throughout the experiment to index changes in cortical excitability. A total of 20 stimulus pulses were delivered, with an interval of 5 s. The EMG signals were filtered and stored in a laboratory computer for offline analysis. To index changes in cortical excitability following rTMS, peak-to-peak MEP amplitudes were averaged across trials before and after each rTMS protocol.

### 2.5. EEG acquisition

Subjects were seated in a reclining armchair with the neck and back supported with a pillow, arms relaxed, and eyes closed. They were asked to avoid eye movements and blinks during recordings. We chose to test subjects with eyes closed to reduce the interference of eye movements and muscle artifacts. Electrode montage and placement were set up according to the international 10/20 system. EEG signals were acquired through a 64-channel BrainAmp EEG system (Brain, Brain Products GmbH, Munich, Germany). A 64-channel EEG cap was positioned on each subject’s head. The reference electrode was at the FCz site, and the ground electrode was at the FPz site. The impedance for all electrodes was kept below 5 kΩ. EEG was recorded for at least 3 min before and after rTMS in the resting state with the eyes closed. The EEG data were sampled at a frequency of 1,000 Hz and filtered through a 0–200 Hz band-pass filter. Data were subsequently processed offline.

### 2.6. EEG processing

EEG data were processed offline using MATLAB (version 17.0) and EEGLAB toolbox (version 14.1.1). All channels were re-referenced to the common average. Unnecessary electrodes (TP9, TP10, FT9, FT10) were removed. Signal periods that contained large muscular and other nonstereotyped artifacts were then carefully pruned from the signals. Continuous recordings were band-pass filtered between 0.5 and 45 Hz and then notched to remove power-line interference (50 Hz). This data selection was followed by independent component analysis. The components, amplitude topography, frequency spectra, and component time series were inspected to identify eye blinks, eye movements, and heart rhythms (Delorme and Makeig, 2004; Delorme et al., 2007), which were removed. 150 s EEG signals without artifacts were selected manually from each subject’s EEG recording. The band-pass filters were used to extract the alpha (8–13 Hz) frequency bands. Finally, a total of 75 segments, each lasting 2 s, were chosen for data analysis. Subsequent power spectral density and functional connectivity analyses were conducted on these 5 s data segments. Fast Fourier transform was applied to estimate the spectral power density for each electrode.

### 2.7. Phase lag index

The functional connectivity between different brain regions was computed using the phase lag index (PLI; Stam et al., 2007). PLI is an indicator of the asymmetry in the distribution of phase differences between two signals, and it reflects the consistency with which one signal is phase leading or lagging in comparison with another. If the phase difference between two time series is $\Delta \Phi \left(t_{k}\right)\left(k=1\ldots N\right)$, then the PLI can be computed as follows:


$$\text{PLI}=\left|sign\left[\Delta \Phi \left(t_{k}\right)\right]\right|$$

where 〈.〉 is the mean value operator. The value of PLI ranges between 0 and 1. A PLI of 0 indicates either no coupling or coupling with a phase difference centered at approximately 0 mod π, whereas a PLI of 1 indicates perfect phase locking at a value of ΔΦ from 0 mod π. The stronger the nonzero phase locking, the larger the value of PLI. A 59 × 59 channel matrix consisting of the PLI values for each electrode pair was obtained for each subject before and after rTMS.

**Figure 2:**
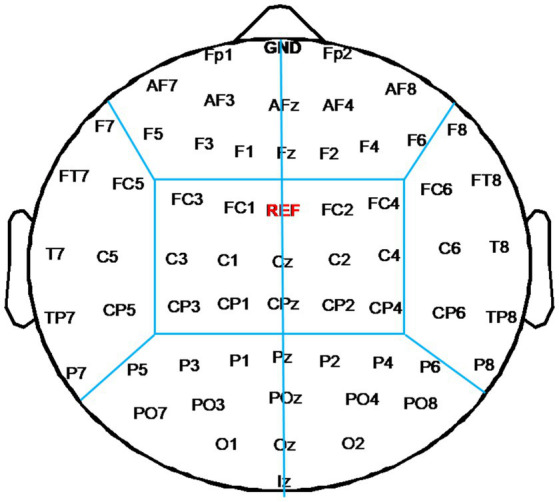
Electrode partition diagram. EEG channels were grouped into eight regions: left and right frontal region, left and right temporal region, left and right central region, left and right occipital region. They are denoted by the letters F, T, C, and T, respectively.

To evaluate changes of functional connectivity induced by each rTMS protocol, EEG electrodes were grouped into eight regions—left frontal region (FP1, AF3, AF7, F1, F3, F5) and right frontal region (Fp2, AF4, AF8, F2, F4, F6), left temporal region (F7, FT7, FC5, C5, T7, CP5, TP7, P7) and right temporal region (F8, FC6, FT8, C6, T8, CP6, TP8, P8), left central region (FC3, FC1, C1, C3, CP1, CP3) and right central region (FC2, FC4, C2, C4, CP2, CP4), left occipital region (P5, P3, P1, PO3, PO7, O1) and right occipital region (P2, P4, P6, PO4, PO8, O2), as shown in Figure 2. In this study, inter-region connections were defined as the mean PLI of the electrode pairs between one region and the other region. The intra-region connections were defined as the mean PLI of the electrode pairs within one region. Midline channels were not used.

### 2.8. Statistical analyses

All data were expressed as mean ± standard deviation. All analyses were performed using SPSS (version 21.0) and MATLAB (version 17.0). Responders and non-responders were classified according to the changes of MEP amplitude, below and above 100% of the baseline, induced by each rTMS protocol. The data normality was tested by the Shapiro–Wilk test for each group. In this study, if changes of each rTMS protocol were normally distributed data, the paired sample *t*-test was used to obtain the changes induced by each rTMS protocol. If the changes of each rTMS protocol were non-Gaussian distributions, the Wilcoxon test was used. For group comparisons of normally distributed data, one-way analysis of variance (ANOVA) was used to obtain the difference between rTMS protocols. *Post hoc* statistics were obtained using Tukey’s method for multiple comparisons. The Kruskal–Wallis test was used for non-Gaussian distributions. The Friedman test with factor “5 rTMS protocols” was applied for MEP amplitude, and Dunn’s method was used for multiple comparisons. The significance level was set at 0.05, unless otherwise indicated.

## 3. Results

All subjects completed the experiments. No subjects reported serious adverse effects during or after the experiments.

### 3.1. Baseline RMT and rTMS intensity

The RMT and rTMS intensity (wearing EEG cap) values at baseline for each rTMS protocol are presented in Table 1. The RMT was about 75% machine output. The rTMS intensity in this study was 80% RMT, which was about 60% machine output. One-way ANOVA confirmed that there were no differences in baseline RMT or rTMS intensity among the five groups (*F* = 0.022, *p* = 0.999).

**Table 1:** RMT and rTMS intensity at baseline.

	Sham	TD: 2 s, ITI: 25 s	TD: 5 s, ITI: 25 s	TD: 5 s, ITI: 50 s	TD: 5 s, ITI: 100 s
RMT	75.00% ± 6.89%	75.33% ± 6.84%	75.67% ± 6.03%	75.53% ± 6.61%	75.47% ± 6.51%
rTMS intensity	60.00% ± 5.51%	60.27% ± 5.47%	60.53% ± 4.83%	60.43% ± 5.29%	60.37% ± 5.21%

RMT, resting motor threshold; rTMS, repetitive transcranial magnetic stimulation; TD, train duration; ITI, inter-train interval.

### 3.2. MEP amplitude

**Figure 3:**
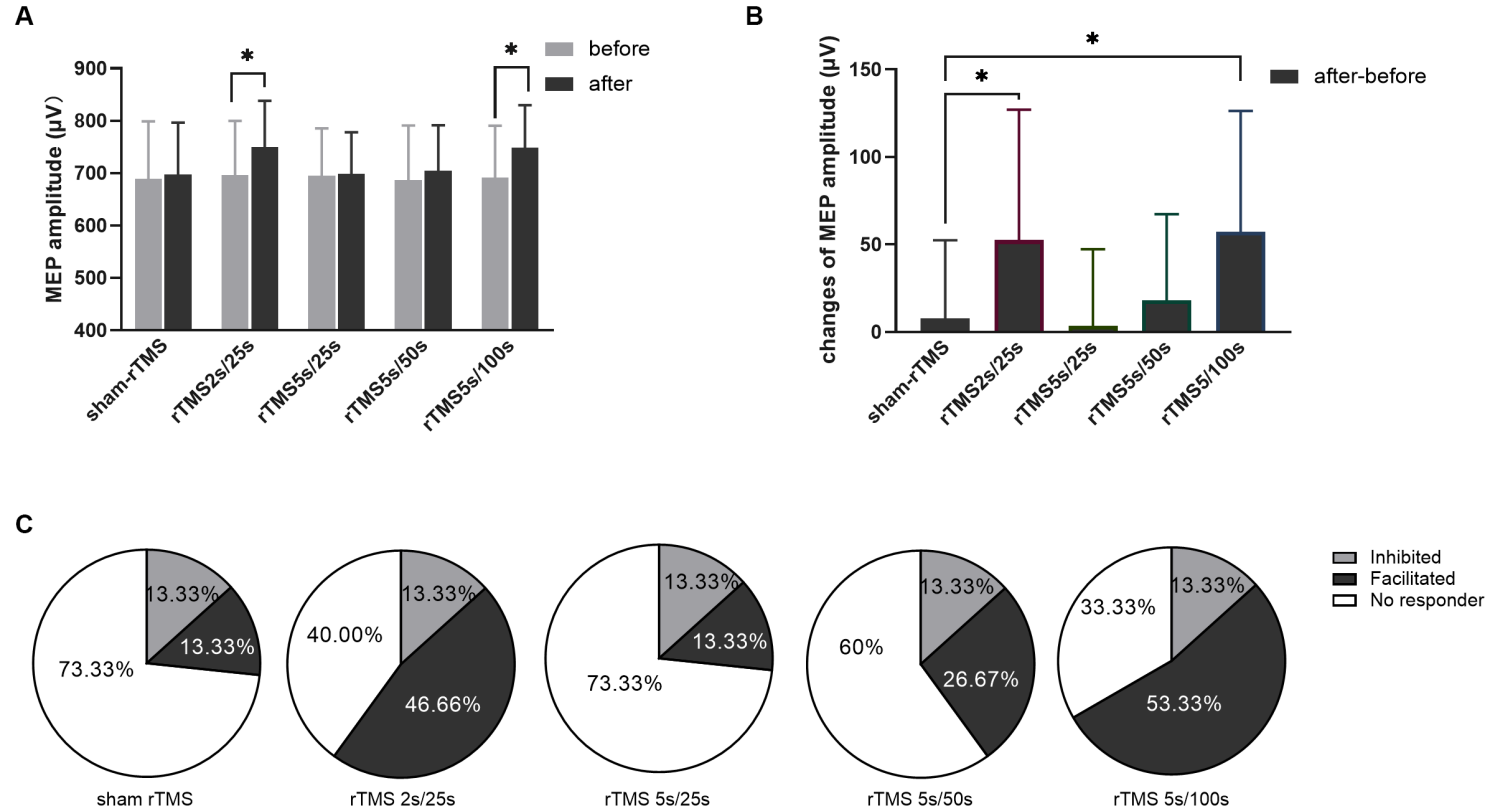
Effects of each rTMS protocol on the MEP amplitude. **(A)** MEP amplitude before and after each rTMS protocol. **(B)** Changes of MEP amplitudes induced by five rTMS protocols. **(C)** Percentage of individuals showing facilitated or inhibited MEPs following each rTMS protocol. Greater than 110% baseline MEP amplitude was counted was as a facilitated response, less than 90% was counted as an inhibited response, and between 90 and 110% was counted as no responders. rTMS2s/25 s, 2 s train duration and 25 s inter-train interval; rTMS5s/25 s, 5 s train duration and 25 s inter-train interval; rTMS5s/50s, 5 s train duration and 50s inter-train interval; rTMS5s/100 s, 5 s train duration and 100 s inter-train interval. The changes of MEP amplitudes with significant differences are marked with stars (*p < 0.05).

The MEP amplitudes were tested before and after each rTMS protocol, and the results are shown in Figure 3. The MEP amplitude did not change after sham rTMS (*t* = −0.6808, *p* = 0.507). The MEP amplitude increased significantly after rTMS2s/25 s (*t* = −2.734, *p* = 0.016). However, when the train duration was prolonged to 5 s, the MEP amplitude did not change (*t* = −0.319, *p* = 0.755). No change was found when the inter-train interval was prolonged to 50 s (*t* = −1.438, *p* = 0.172), but the MEP amplitude increased compared to the baseline when the inter-train interval was prolonged to 100 s (*t* = −3.222, *p* = 0.006). The results showed that 10 Hz rTMS2s/25 s increased the excitability of the corticospinal tract. The effects of 10 Hz rTMS on the excitability of the corticospinal tract disappeared when the train duration was prolonged to 5 s from 2 s but appeared when the inter-train interval was 100 s.

Furthermore, we calculated the changes of MEP amplitude induced by each rTMS protocol through after rTMS minus before rTMS. The changes of MEP amplitude for different rTMS protocols were analyzed using one-way ANOVA. As shown in Figure 3B, different rTMS protocols had significant main effects (*F* = 6.876, *p* = 0.0001). After *post hoc* statistical analysis, the results showed that the changes of MEP amplitude induced by the rTMS2s/25 s and the rTMS5s/100 s were significantly different from those induced by sham rTMS, rTMS5s/25 s and rTMS5s/50s. The changes of MEP amplitude induced by the rTMS5s/25 s and the rTMS5s/50s were not different compared to those induced by sham rTMS. The rTMS2s/25 s was not different compared to the rTMS5s/100 s. The results confirmed that the rTMS2s/25 s and rTMS5s/100 s had a significant influence on the MEP amplitude, whereas the rTMS5s/25 s and rTMS5s/50s did not.

To quantify the percentage of individuals in which MEPs were facilitated or inhibited following each rTMS protocol, the MEP amplitudes were normalized to the baseline. Greater than 110% baseline MEP amplitude was counted was as a facilitated response, less than 90% was counted as an inhibited response, and between 90 and 110% was counted as no responders (Nettekoven et al., 2015; Thomson et al., 2019; Tiksnadi et al., 2020; Bakulin et al., 2022). As shown in Figure 3C, facilitated MEPs were observed in 13.33, 46.67, 13.33, 26.67, and 53.33% of individuals following sham rTMS, rTMS2s/25 s, rTMS5s/25 s, rTMS5s/50s, and rTMS5s/100 s, respectively.

### 3.3. Power spectral density

**Figure 4:**
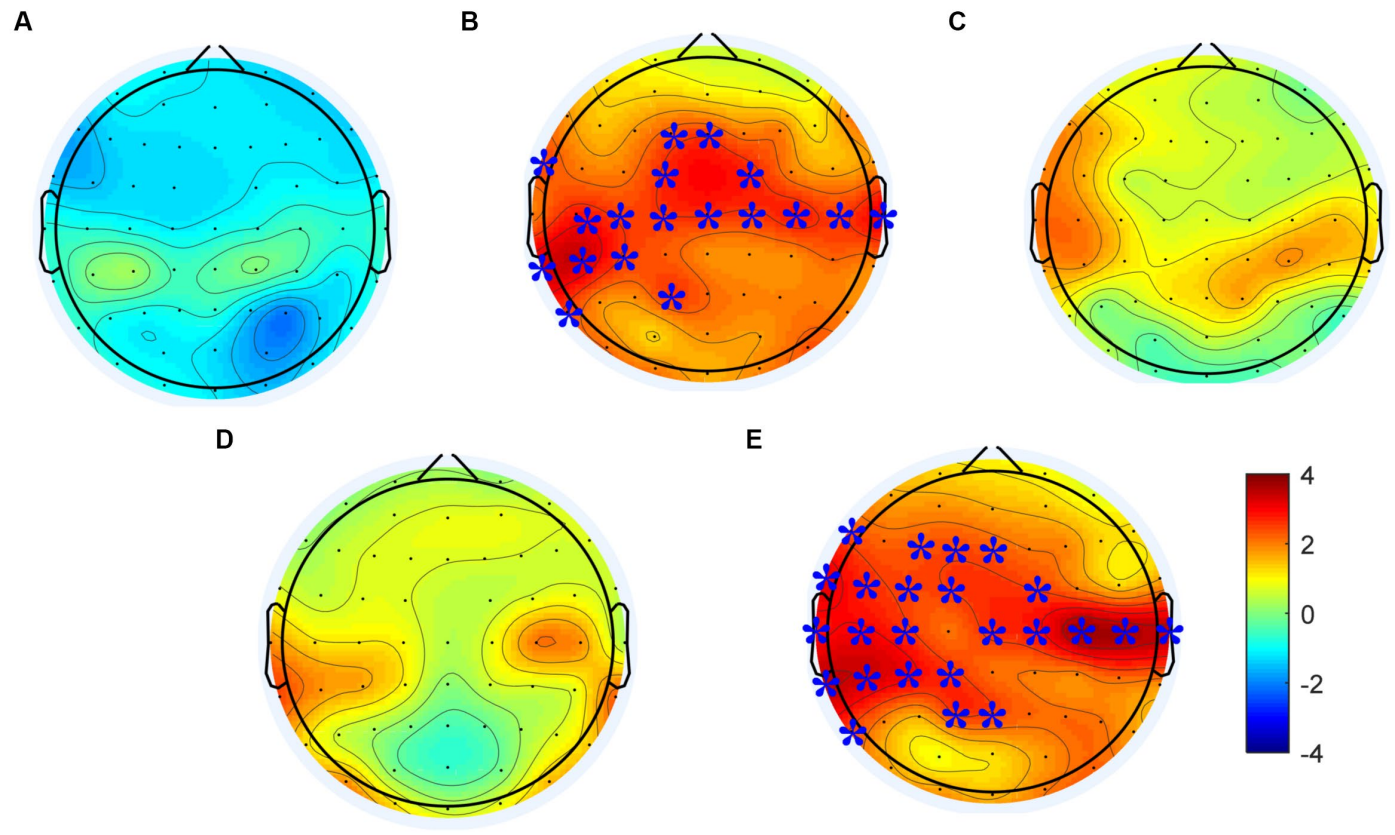
Changes in power spectral density in the alpha frequency band induced by each rTMS protocol. **(A)** sham-rTMS; **(B)** rTMS2s/25 s; **(C)** rTMS5s/25 s; **(D)** rTMS5s/50s; **(E)** rTMS5s/100 s. rTMS2s/25 s, 2 s train duration and 25 s inter-train interval; rTMS5s/25 s, 5 s train duration and 25 s inter-train interval; rTMS5s/50s, 5 s train duration and 50 s inter-train interval; rTMS5s/100 s, 5 s train duration and 100 s inter-train interval. Electrodes showing significant differences are marked with blue stars (*p < 0.01).

Changes of power spectral density in the alpha frequency band (8–12 Hz) were calculated following each rTMS protocol, the results are shown in Figure 4. Electrodes showing significant differences are marked with blue stars (*p* < 0.01). The power spectral density in the alpha frequency band increased after all active (i.e., not sham) rTMS protocols in most brain regions. The power spectral density in the central and temporal regions of both hemispheres increased significantly after the rTMS2s/25 s. Also, the power spectral density in the central and temporal regions of both hemispheres and in the frontal and parietal-occipital junctions of the stimulated hemisphere increased significantly after the. Compared to the rTMS2s/25 s, the changes of power spectral density induced by rTMS5s/100 s were wider in the stimulated hemisphere. The power spectral density increased after the rTMS5s/25 s and after the rTMS5s/50s, but no statistically significant differences were found in any regions. No change was found to be significant following the sham rTMS protocol.

**Figure 5:**
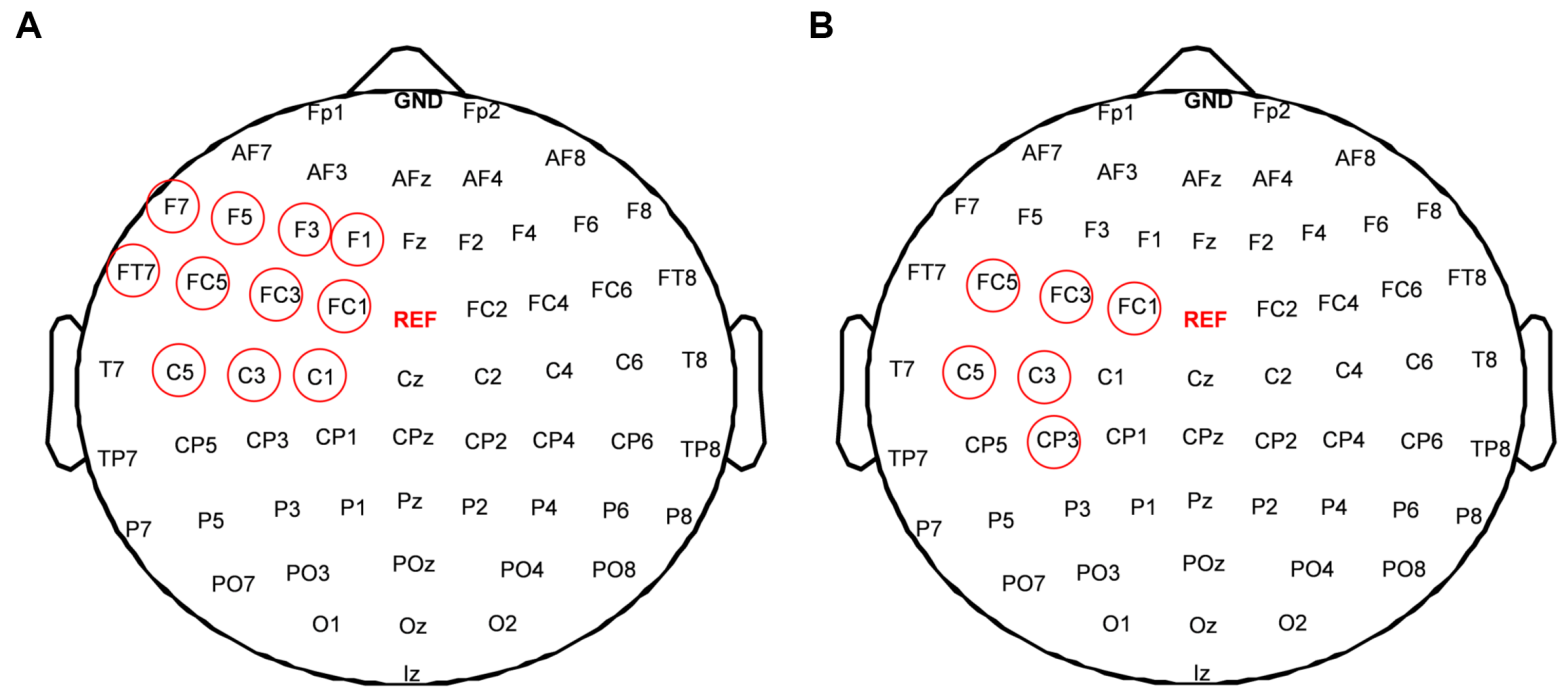
Difference of power spectral density in alpha frequency band among five rTMS protocols; **(A)** between the rTMS2s/25 s and sham-rTMS; **(B)** between the rTMS5s/100 s and sham-rTMS. rTMS2s/25 s, 2 s train duration and 25 s inter-train interval; rTMS5s/25 s, 5 s train duration and 25 s inter-train interval. Red circles show the electrodes which showed the significant difference of power spectral density between two rTMS protocols (*p < 0.01).

The difference of power spectral density in the alpha frequency band among five rTMS protocol were analyzed to assess the different effects of rTMS protocols. The results were shown in Figure 5. Significant effects were observed in the frontal, central, and temporal regions of the stimulated hemisphere for the alpha frequency band (*p* < 0.05). After *post hoc* statistical analysis, the results showed that the power spectral density induced by the rTMS2s/25 s and rTMS5s/100 s was significantly different from that induced by the sham rTMS, which was distributed in the frontal and central regions of the stimulated hemisphere. The power spectral density induced by the rTMS5s/100 s inter-train interval was distributed in a broader brain region, compare to the rTMS2s/25 s, which was distributed in the central and frontal regions of the stimulated hemisphere. There were no significant differences between other active rTMS protocols and the sham rTMS protocol or among active rTMS protocols. The results confirmed that the rTMS2s/25 s and the rTMS5s/100 s had a significant influence on the power spectral density in the alpha frequency band, whereas the rTMS5s/25 s and the rTMS5s/50s did not.

### 3.4. Functional connectivity

**Figure 6:**
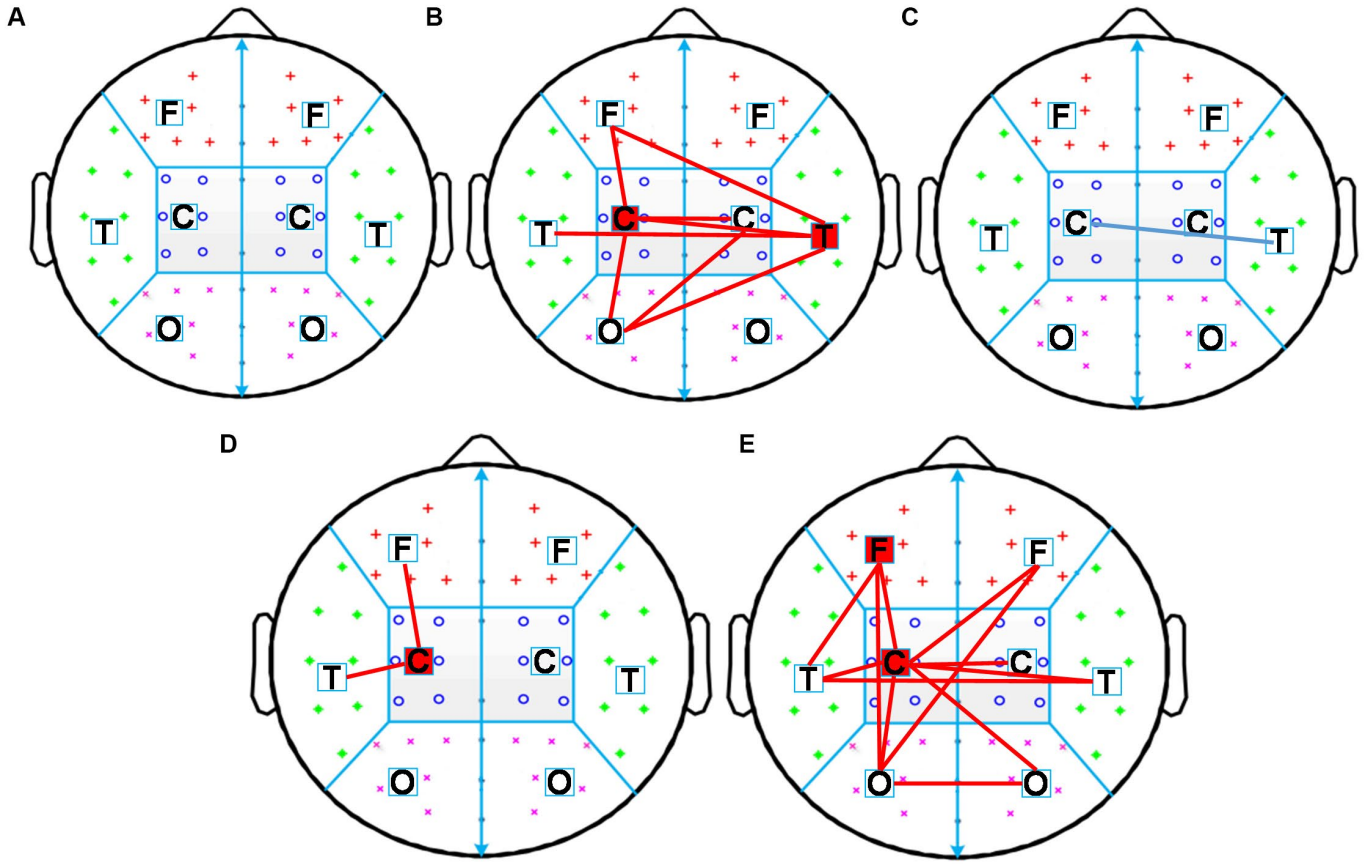
Changes in inter-region and intra-region functional connectivity induced by five rTMS protocols. Inter-region connections is defined as the mean PLI of the electrode pairs between one region and the other region. The intra-region connections is defined as the mean PLI of the electrode pairs within one region. Midline channels were not used. **(A)** sham-rTMS; **(B)** rTMS2s/25 s; **(C)** rTMS5s/25 s; **(D)** rTMS5s/50s; **(E)** rTMS5s/100 s. rTMS2s/25 s, 2 s train duration and 25 s inter-train interval; rTMS5s/25 s, 5 s train duration and 25 s inter-train interval; rTMS5s/50s, 5 s train duration and 50 s inter-train interval; rTMS5s/100 s, 5 s train duration and 100 s inter-train interval. A significant increase in inter-and intra-regions is indicated by red lines and red blocks, respectively. A significant decrease in inter-and intra-regions is indicated by blue lines and blue blocks, respectively.

First, the intra-region PLI values of each region and the inter-region PLI values between all regions in the alpha frequency band were calculated, then significant changes of inter-region and intra-region PLI in the alpha frequency band induced by each rTMS protocol were assessed. The results were shown in Figure 6. The sham-rTMS (Figure 6A) and rTMS5s/25 s (Figure 6C) did not change the PLI in the alpha frequency band. When the inter-train interval was prolonged from 25 s to 50 s, the PLI increased in the central region of the stimulated hemisphere, the inter-regions between the central region and frontal region, and the temporal region of the stimulated hemisphere (Figure 6D). For the rTMS2s/25 s (Figure 6B) and rTMS5s/100 s (Figure 6E), the functional connectivity increased in lots of intra-regions and inter-regions. For the rTMS2s/25 s, the PLI of the inter-regions in the alpha frequency band increased in the central region of the stimulated hemisphere, the inter-regions between the central region of the stimulated hemisphere and the frontal and occipital regions of the stimulated hemisphere, and between the central regions of both hemispheres. The rTMS5s/100 s affected not only the long functional connectivity of inter-regions but also the short functional connectivity of intra-regions in the alpha frequency band, mainly between the central region of the stimulated hemisphere and other regions. More specifically, the rTMS5s/100 s changed the functional connectivity in the central region of the stimulated hemisphere, the connectivity between the central region of the stimulated hemisphere and the frontal and temporal regions of the stimulated hemisphere, and the connectivity between the occipital regions of both hemispheres.

## 4. Discussion

Our study found that 10 Hz rTMS2s/25 s could increase cortex excitability and the power spectral density of bilateral central regions in the alpha band and enhance the functional connectivity between the central regions and other brain regions. When the train duration was prolonged to 5 s, the after-effects of high-frequency rTMS disappeared, and there was no change in any brain region or intra-regions or inter-regions. Furthermore, when the train duration was 5 s and the inter-train interval was extended to 100 s from 25 s, high-frequency rTMS had the same effect as the rTMS2s/25 s. Our results suggested that a single train might be modulated by homeostatic metaplasticity, while a certain length of inter-train interval might make the stimulation train break through the modulation of homeostatic metaplasticity. The train duration and inter-train interval significantly affected the after-effect of high-frequency rTMS.

### 4.1. The effect of train duration on brain activities

It was reported that high-frequency rTMS could produce different effects with different stimulation durations, which was consistent with our findings. Rothkegel et al. found that continuous rTMS could not improve the cortical excitability, and thought the intervals during rTMS were essential for facilitatory after-effects (Rothkegel et al., 2010). Jung et al. investigated the changes in cortical excitability of the human motor cortex induced by high-frequency TMS of different stimulation durations, and found high-frequency rTMS with 5 s train duration could not improve the cortical excitability. The results of those study were inconsistent with the traditional viewpoint that high frequency rTMS can improve cortical excitability (Houdayer et al., 2008; Di Lazzaro et al., 2011). To date, few studies have focused on the after-effect of train duration in high-frequency rTMS.

In recent years, several studies had investigated the modulation of homeostatic metaplasticity induced by rTMS, using priming stimulation combined with testing stimulation methods (Gamboa et al., 2011; Kramár et al., 2012; Opie et al., 2017). These studies used priming stimulation to change the excitability of the cerebral cortex and the testing stimulation to evaluate the effect of rTMS on cortical excitability. The results demonstrated that the after-effect of rTMS was related to the state of brain activity before rTMS stimulation.

In our study, we speculated that, the single long stimulation train, might be understood as two consecutive short stimulation trains, where the first short stimulation train is a priming stimulation to modulate brain activities to a certain state, and the second short stimulation train is a testing stimulation, the after-effects of which are modulated by homeostatic metaplasticity and, thus, prolonging the train duration might not induce changes in brain activity. We selected 10 Hz rTMS with 2 s or 5 s train duration. We found that high frequency rTMS with 2 s train duration can increase the amplitude of MEP, the power spectral density of alpha band and functional connectivity. This is consistent with the traditional viewpoint that high frequency rTMS can improve cortical excitability (Houdayer et al., 2008; Di Lazzaro et al., 2011). However, when the train duration was extended to 5 s, high-frequency rTMS not only did not change the amplitude of MEP, but also did not significantly change the EEG power spectrum and brain interval connections. This confirms our hypothesis (1) prolonging the train duration of high-frequency rTMS might result in the disappearance. The results of this study were consistent with the modultaion characteristics of the homeostatic metaplasticity.

### 4.2. The effect of inter-train interval on brain activities

In high frequency rTMS, after a certain number of pulse stimulation, often set a period of time interval, in order to prevent coil heating and safety consideration. Later, it was found that the inter-train intervals were incorporated into high-frequency rTMS protocols not only to avoid overheating and for safety purposes but also to impact the characteristics of the central nervous system. Rothkegel et al. (2010) found that intervals during 5 Hz rTMS were essential for facilitatory after-effects, and cortical excitability could not be improved by continuous high-frequency rTMS without inter-train interval (Rothkegel et al., 2010). Cash et al. (2017) studied the excitatory and disinhibitory effect of 20 Hz rTMS modulated by different inter-train interval, and found the changes in MEPs did not depend on the inter-train interval, but shorter inter-train interval resulted in greater disinhibitory effects. In our study, we selected 10 Hz rTMS with train duration of 25 s, 50s and 100 s, and found that brain activities, including MEP, power spectral density and functional connectivity, did not change significantly with inter-train intervals of 25 s and 50s. When the inter-train interval was extended to 100 s, not only the MEP amplitude increased, but also the power spectral density and functional connection were significantly changed, compared with the sham group. Our study again demonstrated the importance of inter-train interval in high frequency rTMS.

There might be two possible reasons about the improvement by prolonging the inter-train interval to 100 s. First, neurons firing produces the action potential, which is followed by hyperpolarized postpotential. A series of action potentials have a cumulative effect on the amplitude of hyperpolarized postpotentials, and the duration of the hyperpolarized postpotentials lengthens with the increase of the number of action potentials (Yarom et al., 1985). TMS causes the neuron to depolarize, which is followed by hyperpolarization. With the increase of the number of TMS pulses, the number of action potentials increased, and the amplitude and duration of hyperpolarization increased. Second, a long enough time must pass for high-threshold synapses to be activated and have an effect. That is, because no interval or a short interval is not enough to reduce the threshold of high-threshold synapses, resulting in the enhancement of high-threshold synaptic plasticity. On the other hand, in synapses with different plasticity thresholds, the activation of synaptic mechanisms and protein synthesis required for hard-to-induce LTP effects takes a sufficiently long time (Kramár et al., 2012). In the study of TMS, the tradeoff between train duration and inter-train interval needs to be further studied.

### 4.3. rTMS changed the activities of alpha rhythm

The alpha rhythm in the sensorimotor cortex is the most specific rhythm of this cortex in the resting state (Hari, 2006). In our study, the power spectral density in the alpha frequency band increased when induced by high-frequency rTMS, a finding consistent with existing views: alpha generation may represent an intrinsic induced response and a basic signature response to TMS targeting the human resting motor cortex (Veniero et al., 2011; Jin et al., 2019). Alpha frequency oscillation (8–13 Hz) in the resting state is an important neural substrate for cognition and motor function. Brain activity in the alpha frequency band can predict the efficiency of cognitive, motor, and other neural processes (Klimesch, 1997; Klimesch et al., 2003; Babiloni et al., 2010). It might be possible to predict the changes of brain efficiency with the changes of train duration and inter-train interval, according to the alpha frequency band. In our study, the results suggested that the changes in the power spectral density and functional connectivity of resting alpha band might reflect whether the brain was in homeostatic metaplasticity moduation.

A number of studies had demonstrated that rTMS over primary motor cortex could alter the activity and function of targeted brain region as well as its related remote regions (Jing and Takigawa, 2000; Plewnia et al., 2008; Grefkes et al., 2010). In our study, we found that there was no significant difference in the amplitude of MEP induced by rTMS2s/25 s and rTMS5s/100 s (Figure 3). However, we found that compared to sham rTMS, rTMS2s/25 s and rTMS5s/100 s increased the power spectral density and functional connectivity in alpha frequency band, and rTMS5s/100 s caused the increase in a wider range of brain regions (Figures 4, 5). This suggested that rTMS5s/100 s might modulate the brain better, in the form of affecting multiple brain regions and brain networks. The activity of many cortices related to motor function, such as premotor, primary motor, and posterior parietal cortex (Youssofzadeh et al., 2016; De Vico Fallani et al., 2017), and the functional connectivity between multi-regions reflects the functional interactions between the underlying brain regions (Ward and Cohen, 2004; Grefkes et al., 2010). Maybe, rTMS5s/100 s might be a more effective protocol to improve the motor function, which is worthy of further study in the future.

### 4.4. The contribution of this study and future directions

It was found in our study that the excitatory effect of high-frequency rTMS disappeared when the train duration was prolonged, but it reappeared when the inter-train interval was prolonged to a specific value, that is, when the excitatory effect of high-frequency rTMS was increased, if a longer train duration was used, a longer inter-train interval was required. If only the train duration was increased without a corresponding increase in inter-train interval, the modulation effect on the brain activities may reach the threshold of no response, or even exceed the threshold of long-term potentiation, and make the effect of rTMS reverse. Therefore, there is a trade-off between train duration and inter-train interval, which influences the effects of high-frequency rTMS.

First, our study suggested that the train duration and inter-train interval should be taken into account when discussing stimulation protocols in both research and clinical practice. More importantly, since the effect of high-frequency rTMS on the brain activities is the superposition of multiple trains, and each train is composed of multiple magnetic pulses. We speculated that for the train duration of rTMS, the neuron excitation state gradually increased with the increase of the number of rTMS stimulation pulses, and the neuron excitation state reached the highest value when the pulses number increased to a certain number. At this time, if the pulse output continued, the neuron excitation began to decrease or even reverse under the influence of homeostatic metaplasticity mechanism. Our study suggested that the brain-state stimulation rTMS methods based on homeostatic metaplasticity would be the future directions, in order to improve the effects of rTMS.

In our study, the power spectral density and functional connectivity induced by rTMS with different train duration and inter-train interval were different, which suggested that in the future, we might be able to use power spectral density or functional connectivity as a sign of homeostatic metaplasticity. That is, we might judge the brain-state through the changes of power spectral density or functional connectivity, and decide whether to continue rTMS pulse output.

Our results suggested that the effects of rTMS with different the train duration and inter-train interval were different, which might be influenced by the mechanism of homeostatic metaplasticity. Similarly, we belive that when rTMS was performed over other brain regions, that the homeostatic metaplasticity also existed. However, it is worth mentioning that the train duration inducing the homeostatic metaplasticity might be different for other brain regions, which needs to be verified by detailed experiments in the future. Furthermore, the results in our study might be used to other stimulation protocols. For example, different TBS and tES protocols might induce the homeostatic metaplasticity, the power spectral density and functional connectivity might be used to optimize the parameters to improve the effects of brain-stimulation.

### 4.5. Limitations

This is the first study describing neural activity changes as a response to prolonged duration of stimulation train in high-frequency rTMS in the same subjects. We ensured that, except for the inter-train interval parameter in experiments 1 and 2, the experimental conditions and TMS parameters were the same for all rTMS protocols to eliminate the impact of all other factors. However, our study has some limitations. First, the number of subjects was small (15 subjects were included in the final analysis), although we performed the experiment 90 times. Studies with a larger sample could help provide more reliable results on the homeostasis mechanism, which was reduced by prolonging the duration of stimulation trains. Despite these limitations, the results confirm that rTMS with different inter-train intervals can induce different oscillatory brain activity. Second, in our study, the persistent post-rTMS effects were not measured, but we measured neural oscillations across the brain using EEG and still found no changes in activity induced by prolonged duration of stimulation train of high-frequency rTMS. Third, in our study, MEPs were collected when the hand muscles were relaxed, which was the most commonly used method to detect motor cortical excitability. MEPs collected during muscle contraction is another method that can be used to assess the motor cortical excitability and will be considered in the future.

## 5. Conclusion

Our study showed that the high-frequency rTMS with a longer train duration could trigger the modulation of homeostatic metaplasticity, which was reflected by the MEP amplitude, power spectral density, and functional connectivity before and after rTMS. Furthermore, a longer interval in rTMS might break the modulation of homeostatic metaplasticity. Our results indicated that train duration and inter-train interval significantly affected the after-effect of high-frequency rTMS. Clinicians and researchers should not only use stimulation frequency but also train duration and inter-train interval when performing rTMS stimulation treatments and experiments to classify the after-effect of rTMS.

## Data availability statement

The raw data supporting the conclusions of this article will be made available by the authors, without undue reservation.

## Ethics statement

The studies involving human participants were reviewed and approved by the Ethics Committee of the Institute of Biomedical Engineering, Chinese Academy of Medical Sciences and Peking Union Medical College. The patients/participants provided their written informed consent to participate in this study.

## Author contributions

JJ, XW, HW, ZL, and TY designed the study. JJ and XW conducted the study. HW and XW recruited subjects. JJ conducted the statistical analyses and drafted the manuscript, which all authors critically revised. All authors contributed to the article and approved the submitted version.

## Funding

This study was supported by the National Natural Science Foundation of China (grant number: 52007199, 81927806), the China Key Research and Development Program (grant number: 2022YFC2402202), and the CAMS Initiative for Innovative Medicine (grant number: 2021-I2M-1-058).

## Conflict of interest

The authors declare that the research was conducted in the absence of any commercial or financial relationships that could be construed as a potential conflict of interest.

## Publisher’s note

All claims expressed in this article are solely those of the authors and do not necessarily represent those of their affiliated organizations, or those of the publisher, the editors and the reviewers. Any product that may be evaluated in this article, or claim that may be made by its manufacturer, is not guaranteed or endorsed by the publisher.
