# Supplementary material for: Train duration and inter-train interval determine the direction and intensity of high-frequency rTMS after-effects
Source: Front Neurosci. 2023 Jul 5;17:1157080. doi: 10.3389/fnins.2023.1157080 (PMC10355321; doi:10.3389/fnins.2023.1157080)
Supplement: Supplementary file 1 [file Data_Sheet_1.docx]

**Supplementary Material**

**The effect of rTMS with different train duration and inter-train interval on beta frequency band**

1. **Extracting the beta frequency band**

150 s EEG signals without artifacts were selected manually from each subject’s EEG recording. The band-pass filters were used to extract the beta (13–30 Hz) frequency band. Finally, a total of 75 segments, each lasting 2 s, were chosen for data analysis. Subsequent power spectral density and functional connectivity analyses were conducted on these 5-s data segments. The power spectral density and functional connectivity were caculated, using the same methods of the main body in manuscript.

1. **Results**

**2.1 Power spectral density**

We found the changes in power spectrum density induced by rTMS2s/25s and rTMS5s/100s in beta frequency bands increased not only in the stimulated hemisphere but also in the remote non-stimulated hemisphere. The power spectral density in the central regions of both hemispheres increased significantly after the rTMS2s/25s. Compared to the rTMS2s/25s, the changes of power spectral density induced by rTMS5s/100s were wider, including in the central and temporal regions of both hemispheres. No significant change was found after the sham-rTMS, rTMS5s/25s and rTMS5s/50s.

Figure 1S. Changes in power spectral density in the beta frequency band induced by each rTMS protocol. (A) sham-rTMS; (B) rTMS2s/25s; (C) rTMS5s/25s; (D) rTMS5s/50s; (E) rTMS5s/100s.

Notes: rTMS2s/25s: 2s train duration and 25s inter-train interval; rTMS5s/25s: 5s train duration and 25s inter-train interval; rTMS5s/50s: 5s train duration and 50s inter-train interval; rTMS5s/100s: 5s train duration and 100s inter-train interval. Electrodes showing significant differences are marked with blue stars (*p<0.01).

**2.2 Functional connectivity**

First, the intra-region PLI values of each region and the inter-region PLI values between all regions in the alpha frequency band were calculated, then significant changes of inter-region and intra-region PLI in the alpha frequency band induced by each rTMS protocol were assessed. The results were shown in Figure S2. The sham-rTMS (Figure S2A) did not change the PLI in the alpha frequency band. For rTMS5s/25s, the PLI of inter-regions in beta frequency band decreased between central and frontal region. Interestingly, rTMS2s/25s and rTMS5s/100s changed the functional connectivity in the central region of the stimulated hemisphere, and the inter-regions functional connectivity between the central region of the stimulated hemisphere and other region of the stimulated hemisphere.

Figure 2S Changes in inter-region and intra-region functional connectivity induced by five rTMS protocols. Inter-region connection was defined as the mean PLI of the electrode pairs between one region and the other region. The intra-region connections is defined as the mean PLI of the electrode pairs within one region. Midline channels were not used. (A) sham-rTMS; (B) rTMS2s/25s; (C) rTMS5s/25s; (D) rTMS5s/50s; (E) rTMS5s/100s. Notes: rTMS2s/25s: 2 s train duration and 25 s inter-train interval; rTMS5s/25s: 5 s train duration and 25 s inter-train interval; rTMS5s/50s: 5 s train duration and 50 s inter-train interval; rTMS5s/100s: 5 s train duration and 100 s inter-train interval. A significant increase in inter- and intra-regions is indicated by red lines and red blocks, respectively. A significant decrease in inter- and intra-regions is indicated by blue lines and blue blocks, respectively.
